# Genetic alterations in Wnt family of genes and their putative association with head and neck squamous cell carcinoma

**DOI:** 10.5808/gi.20065

**Published:** 2021-03-26

**Authors:** Jain Aditya, A. S. Smiline Girija, A. Paramasivam, J. Vijayashree Priyadharsini

**Affiliations:** 1 Department of Microbiology, Saveetha Dental College, Saveetha Institute of Medical and Technical Sciences (SIMATS), Saveetha University, Chennai 600077, India; 2 Biomedical Research Unit and Laboratory Animal Centre-Dental Research Cell, Saveetha Dental College, Saveetha Institute of Medical and Technical Sciences (SIMATS), Saveetha University, Chennai 600077, India

**Keywords:** gene amplification, genetic variants, head and neck squamous cell carcinoma, in silico, Wnt pathway

## Abstract

Head and neck squamous cell carcinoma (HNSCC) is the most frequent type of head and neck cancer that usually arises from the mucosal surfaces of several organs including nasal cavity, paranasal sinuses, oral cavity, tongue, pharynx, and larynx. The Wnt signaling pathway is a crucial mechanism for cellular maintenance and development. It regulates cell cycle progression, apoptosis, proliferation, migration, and differentiation. Dysregulation of this pathway correlates with oncogenesis in various tissues including breast, colon, pancreatic as well as head and neck cancers. The present study aims to assess the gene alterations in the Wnt family of genes so as to derive an association with HNSCC. Computational approaches have been utilized for the identification of gene alterations in the Wnt family of genes. Several databases such as cBioportal, STRING, and UALCAN were used for the purpose. The frequency of alteration was high in case of Wnt family member 11 (5%). Gene amplification, deep deletions, missense and truncating mutations were observed in HNSCC patients. There was a marked difference in the gene expression profile of WNT11 between grades as well as normal samples. The survival probability measured using the Kaplan-Meier curve also presented with a significant difference among male and female subjects experiencing a low/medium level expression. The female patients showed less survival probability when compared to the male subjects. This provides the prognostic significance of the *WNT11* gene in HNSCC. Taken together, the present study provides clues on the possible association of *WNT11* gene alterations with HNSCC, which has to be further validated using experimental approaches.

## Introduction

The head and neck squamous cell carcinoma (HNSCC) represents a heterogeneous group of cancer affecting the mucosal surfaces of several organs including nasal cavity, paranasal sinuses, oral cavity, tongue, pharynx, and larynx [1]. HNSCC accounts for more than 330,000 deaths worldwide with more than 650,000 cases of HNSCCs reported annually [2]. The development of HNSCC is strongly associated with long-term tobacco use, excessive consumption of strong alcohols or, especially in the case of oropharyngeal tumors, the infection with human papillomavirus (HPV), usually HPV type 16 or 18 [3]. The incidence of HNSCC is high in males when compared to females, especially in eastern Europe and India with over 20 males affected per 100,000 individuals [4]. Despite relatively easy access for clinical inspection, these tumors are frequently detected at a late stage, when therapeutic options are less effective in curing patients, who are then at a greater risk of the development of recurrent tumors or metastasis [5]. Thus, the overall survival rates in this group of patients remain relatively low (~50%), especially when patients are diagnosed with advanced stages of the disease [6]. There is a need for novel biomarkers which could improve the clinical management of HNSCC, including better prognosis and disease monitoring. Moreover, the development of new therapeutic options is also necessary for the improvement of treatment outcomes.

Wnt signaling is vital for a plethora of cellular function ranging from homeostasis to the development of mature tissue. Embryonic development also requires Wnt-mediated canonical signaling [7-9]. Moreover, Wnt signaling is inevitable for regulating cellular proliferation, apoptosis, metastasis, and migration of cells [10]. Of note, Wnt operates through either canonical or non-canonical pathways which are differentiated by β-catenin involvement [11]. Cell cycle progression, differentiation, fate determination, and migration are generally orchestrated by canonical *Wnt* signaling. Altered Wnt/β-catenin signaling has been considered a promoting event for various types of cancers and the oncogenic potential of Wnt signaling has been discussed in numerous cancer types, including breast, pancreatic, colon as well as head and neck [12]. The present study investigates the genetic alteration within the Wnt family genes employing in silico approach. The study is first of its kind that reports frequency and type of mutation in genes of the Wnt signaling pathway which provides a clue on the putative association of these genes with HNSCC.

## Methods

### Sample data set

The data is obtained from the cBioportal database which is a web source for obtaining, analyzing and exploring genomes. It contains the description of patients from different cohorts and provides information on the genetic alterations among various samples and genes. The Cancer Genome Atlas, TCGA (Firehose Legacy) data set consists of a total of 528 cases of head and neck squamous cell carcinoma, of which 504 samples had sequencing and copy number alteration data. The complete profile of mutated, amplified, deleted genes for each sample has been recorded in the database. Table 1 contains the demographic data of the patients analyzed in the study. Oncoprint data was obtained on submitting user-defined queries on 19 genes of the Wnt family, which was further analyzed for gene expression profile.

### Oncoprint data analysis

**Fig. 1.**
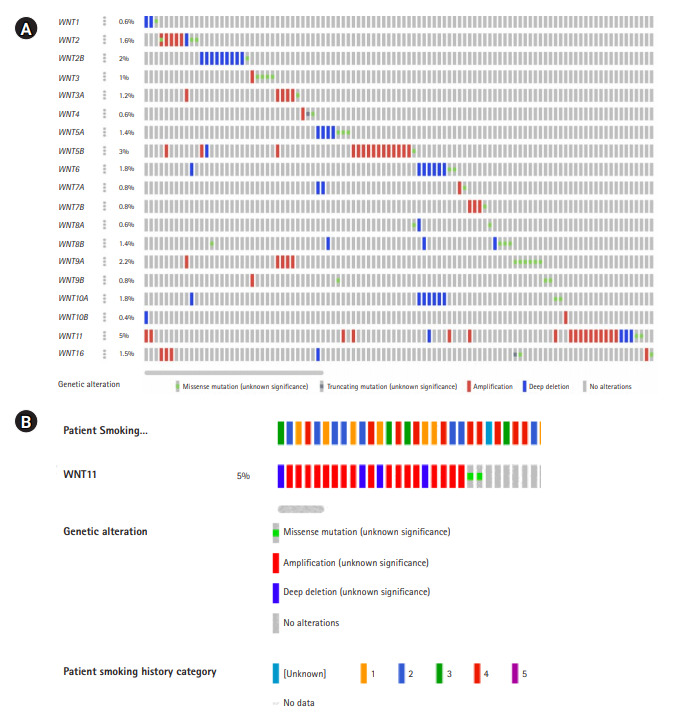
(A) Oncoprint analysis depicting gene alterations in the Wnt family of genes. Each of the grey bars represent patients with head and neck squamous cell carcinoma. (B) Frequency of gene amplification among different categories of smokers.

Oncoprint analysis is the shortened and concise summation of the genetic alterations in graphical format. It provides data on gene alterations based on user-defined query on a specific gene or a gene family. The details on frequency distribution of variations in each of the genes, the variant allele frequency, gene deletions, amplifications, insertions, frameshift etc., were recorded (Fig. 1) [13,14]. These information were used as baseline to track mutations or variations, gene expression, and survival of patients based on the gene alterations using several other computational tools.

### gnomAD analysis

The genome aggregation database (gnomAD) hosts information on 125,748 exome sequences and 15,708 whole genome sequences from unrelated individuals sequenced and deposited as part of various disease-specific or population genetic studies. The data source obtained from oncoprint was used to identify whether the variations observed in the present study were novel or reported elsewhere in any other population (Table 2). The exhaustive data source also provides information on minor allele frequencies which will provide a clue as whether the variant identified is a mutation or a polymorphism [15].

### Protein protein interaction network analysis

The STRING database is a collection of known and predicted protein-protein interactions. These interactions could either be direct (physical) and indirect (functional) associations. They are derived from computational predictions and text mining of protein interactions in different organisms and information aggregated from several other primary databases [16]. The gene exhibiting the highest frequency of gene alteration was selected from the entire family and investigated for gene expression and derivation of expression based survival curves for different combinations of parameters such as sex, ethnicity, tumor grade etc., Functional enrichment of the protein network and Kyoto Encyclopedia of Genes and Genomes pathway was derived from the protein protein interaction (PPI) network. The strength score is the ratio between the number of proteins annotated with a term interacting in the network and the number of proteins which is expected to be annotated with this term in a random protein network of the same size. The false discovery rate demonstrates the significance of the enrichment process. The false discovery rate is denoted by p-values which are corrected for multiple testing within each category using Benjamini-Hochberg procedure.

### Gene expression and survival analysis

The expression of the gene presenting with highest frequency of gene alteration in HNSCC was analyzed using the UALCAN (http://ualcan.path.uab.edu/cgi-bin/TCGA-survival) database. Survival curve analysis based on the tumor grade and expression profile was performed to demonstrate the putative role of Wnt family of genes with HNSCC. Gene expression data is expressed as transcripts per million (TPM) which is a normalization method for RNA-sequencing data. The TPM values which were used for the generation of box-whisker plots were also used to determine the significant difference between the groups. The t test was performed using PERL script with the comprehensive Perl archive network (CPAN) module. Combined survival effect analysis of gene expression and other clinical parameters such as race, sex, tumor grade, and cancer subtypes were assessed using log-rank test that generated a p-value which was further used to indicate statistical significance of survival correlation between groups [17].

## Results

### Demographic data

The dataset (TCGA, Firehose Legacy) included in the present study had information on 528 HNSCC samples. The male:female ratio was found to be 2.7:1, with age groups ranging from 19 to 90 years. The number of individuals with the history of smoking and alcohol were roughly around 98% and 67%, respectively. There were five different groups of categories for smoking viz., 1-lifelong non-smoker, 2-current smoker, 3-current informed smoker for >15 years, 4-current reformed smoker ≤15 years, 5-current reformed smoker, duration not specified. The dataset had samples from patients of American (85.6%), African (9.1%), Asian (2.1%), and American Indian (0.4%) descent. The distribution of patients based on the histologic grade of neoplasm is given in Table 1, of which 59% of patients had grade 2 tumor.

### Oncoprint data analysis

The oncoprint data analysis revealed alterations in 19 genes, of which *WNT11* (5%) harbored the highest frequency of gene amplification and deep deletion. When the pattern of amplification was assessed in different groups of smokers a greater frequency of gene amplification was observed in current smokers (n=6) when compared to other categories (Table 2, Fig. 1B).

### gnomAD analysis

The gnomAD analysis revealed several novel and reported variants as demonstrated by the oncoprint data. The variations in *WNT3* (R85Q), *WNT5A* (I93V, G341S), *WNT6* (R46W, T105M), *WNT7A* (R90C), *WNT10A* (A240V), and *WNT11* (L65P, R202H) genes were reported. Other missense mutations were found to be novel (Table 2). Further investigations are warranted to identify the consequences and association of these variations with HNSCC.

### Protein network analysis

**Fig. 2.**
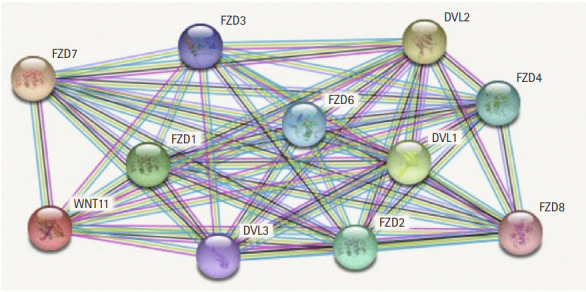
The proteins network interaction of *WNT11* gene.

The protein interaction network reveals the major interactions of *WNT11* with genes such as *DVL1*, *DVL2*, *DVL3*, *FZD1*, *FZD2*, *FZD3*, *FZD4*, *FZD6*, *FZD7*, *FZD8* which play key roles in governing cell polarity, embryonic development, formation of neural synapses, cell proliferation, and many other processes in developing and adult organisms (Fig. 2). Majority of genes interacting with *WNT11* exhibit significant upregulation of the transcripts with *FZD2* and *DVL3* showing a marked difference (p < 10^-12^) in the expression pattern. The expression score is demonstrated by a p-value which denotes the significant difference between two groups of samples viz., normal and HNSCC (Table 3). The functional enrichment analysis showed eleven nodes and 55 edges. The proteins were found to interact more among themselves suggestive of a biologically connected group. The overall PPI enrichment p-value was found to be <1.0 × 10^-16^. Pathways derived from Kyoto Encyclopedia of Genes and Genomes (KEGG) analysis returned predictions which were more inclined towards other cancer types such as basal cell carcinoma, hepatocellular carcinoma, etc. (Table 4).

### Gene expression and survival analysis

**Fig. 3.**
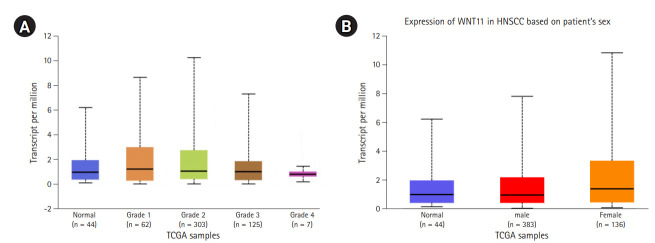
(A) Box-Whisker plot showing relative expression profile of *WNT11* gene in different grades of head and neck squamous cell carcinoma (HNSCC). The X axis denotes The Cancer Genome Atlas (TCGA) samples and Y axis denotes the transcripts per million values. The comparison of gene expression patterns between different grades of HNSCC returned significant values between normal vs grade 2 (p = 3.8 × 10^-2^), normal vs. grade 4 (p = 4.3 × 10^-3^), grade 1 vs. grade 4 (p = 7.11 × 10^-3^), grade 2 vs. grade 4 (p = 2.54 × 10^-11^) and grade 3 vs. grade 4 (p = 1.9 × 10^-3^). A p-value less than 0.05 was considered to be significant. (B) Box-Whisker plot showing relative expression profile of *WNT11* gene in male and female HNSCC subjects. The X axis denotes the TCGA samples and Y axis denotes the transcripts per million values. The comparison of gene expression patterns between male and female viz., normal vs. male (p = 9.923 × 10^-2^), normal vs. female (p = 2.169 × 10^-2^), male vs. female (p = 2.12 × 10-1). A p-value less than 0.05 was considered to be significant.

**Fig. 4.**
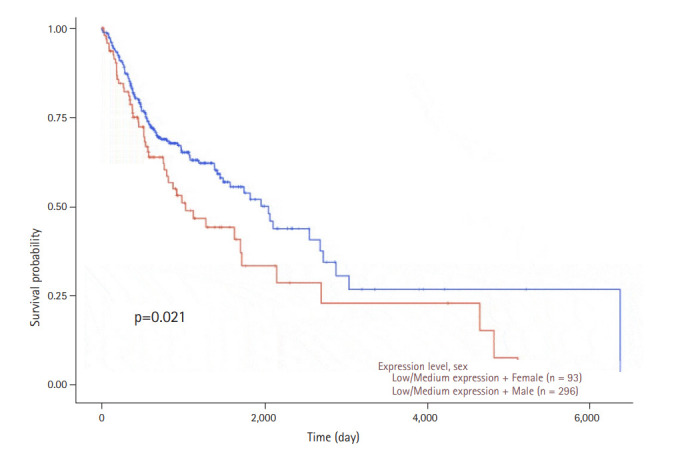
Kaplan-Meier plots showing the association of *WNT11* gene expression in combination with the sex with head and neck squamous cell carcinoma patient’s survival. The x-axis represents time in days and y-axis shows the survival probability. The blue line indicates low/medium expression in male patients and the red line indicates low/medium level expression of the *WNT11* gene in female patients. A significant difference in the survival probability was observed between the two groups (p = 0.021). Female subjects with a low/medium level expression presented with a low survival probability when compared to male subjects. A p-value less than 0.05 was considered to be significant.

The gene expression profile of *WNT11* between normal and primary tumor samples revealed a significant difference with a p-value of 3.043 × 10^-3^. The relative expression profile of *WNT11* gene in different grades of HNSCC also returned significant values between normal vs. grade 2, normal vs. grade 4, grade 1 vs. grade 4, grade 2 vs. grade 4, and grade 3 vs. grade 4 (Fig. 3A). The expression pattern of *WNT11* gene produced significant difference between normal and female HNSCC subjects (p = 2.169 × 10^-2^). Significant difference was not observed between groups normal vs. male and male vs. female (Fig. 3B). Although the present observation do not confirm sex predilection of *WNT11* gene expression with HNSCC, a significant difference in the survival probability between male and female subjects was observed with low/medium level expression (p = 0.021). Furthermore, female subjects who presented with a low/medium level expression exhibited low survival probability when compared to male subjects. A p-value less than 0.05 was considered to be significant (Fig. 4).

The expression profile of *WNT11* was checked in other types of squamous cell carcinoma such as lung (LUSC) and esophageal cancers (ESCC). Although both LUSC and ESCC produced a significant difference with respect to normal and primary tumors, WNT11 expression was upregulated in ESCC cases which was in consonance with HNSCC type. In both the cancer types, there was upregulation of *WNT11* gene, whereas in case of LUSC *WNT11* expression was downregulated. The receptor proteins interacting with *WNT11* have also shown significant differences based on the levels of gene expression in different sex groups (data not shown). These results add to the association of *WNT11* gene alterations with HNSCC.

## Discussion

Genetic variations such as single nucleotide variants and copy number variants have long been associated with oral cancer and other devastating diseases. Wnt signaling is vital for a plethora of cellular function ranging from homeostasis to the development of mature tissue. Embryonic development also requires Wnt-mediated canonical signaling [7–9]. Moreover, Wnt signaling is inevitable for regulating cellular proliferation, apoptosis, metastasis, and migration of cells [10]. Of note, Wnt operates through either canonical or non-canonical pathways which are differentiated by β-catenin involvement [11]. Both pathways are activated by the binding of Wnt protein to the Frizzled (Fzd) seven transmembrane receptor. The fundamental difference between these two pathways is the involvement of β-catenin [18,19].

Non-canonical Wnt signaling pathways, which are independent of β-catenin, rely on the signal transduction of Wnt through Fzd as well as its co-receptors such as receptor tyrosine kinase-like orphan receptor 2 or receptor-like tyrosine kinase [20]. On the other hand, the canonical Wnt signaling pathway, also known as Wnt/β-catenin signaling pathway, involves the activation of cytoplasmic β-catenin signaling cascades upon Wnt signal transduction at the cell membrane [21]. Altered Wnt/β-catenin signaling has been considered a promoting event for various types of cancers. Canonical Wnt signaling pathway acts as a master regulator for a wide range of biological effects through up- or down-regulation of genes that act as direct effectors, transcription regulators, or other signaling pathway regulators. Thus, Wnt target gene expression can either directly or indirectly activate cell cycle progression, cell proliferation, cell differentiation, cell migration, inhibit apoptosis, and regulate embryonic development.

The involvement of canonical Wnt signaling pathway in the formation of HNSCC has also been examined in several experimental studies. Evidence of the association between the pathway and HNSCC was first discovered through cDNA arrays on patient samples. The study found that Fzd, Fzd homolog 3, and Dvl homolog genes, which are functionally important in the canonical Wnt signaling pathway, were highly expressed in HNSCC by two to five fold when compared to normal tissues [22]. Furthermore, another study has also demonstrated that the gene expression levels of Wnts, particularly Wnt11 and Wnt10b, were markedly higher by 17 and 3-fold, respectively, in HNSCC cells compared to normal oral squamous epithelial cells [23]. Over-expression of these major components in HNSCC cells compared to normal cells clearly signifies the involvement of abnormal activation of Wnt signaling cascades in HNSCC. These reports were in agreement with the observation made in the present study wherein the up-regulation of *WNT11* correlated with concomitant increase in the gene expression level of interacting genes. Recently, the role of canonical Wnt signaling pathway in regulating self-renewal of HNSCC cancer stem cells is also being emphasized in several studies. Abnormal activation of the pathway has been correlated with increased proliferation and thus self-renewal of cancer stem cells in HNSCC [24]. These observations were confirmed by our computational analysis wherein the KEGG pathways arising out of functional enrichment analysis revealed involvement of *WNT11* in multiple pathways *viz*., pathways associated with cancer, infections, syndrome, and signaling process regulating pluripotency of stem cells.

Although gene expression profiling is well documented in case of *WNT* family of genes, the effect of gene alterations such as mutations, deletions, and copy number variations upon the expression of the genes in the family is scarce or limited. The present study throws light on those alterations which might act as putative drivers in establishing tumorigenesis. Numerous novel variants and reported variants were identified in HNSCC patients. The potential role of these variants in the disease process is yet to be explored. Similar studies have already been carried out to unravel the potential markers with putative association with HNSCC. The survival curve analysis provides cues on the prognostic significance of these markers in relation to the disease [25,26].

Computational tools provide a cost-effective alternative for analyzing the genetic alterations especially in a complex disorder such as cancer. Although the crosstalk between multiple signaling pathways play a vital role in the development of cancer, further understanding of one important molecular mechanism, which is the canonical Wnt signaling pathway, is critical as targeting the pathway could be a promising approach in eradicating the treatment failure and relapse in HNSCC. With all the pros addressed, the study design also suffers certain drawbacks such as population bias and reproducibility in other ethnic groups. The identification of polymorphic variants and chromosomal abnormalities would aid us in preparing a panel of markers intended for use as early diagnostic leads. These markers can be further validated using genotyping methods or next-generation sequencing approaches to derive a strong association with the disease phenotype.

## Figures and Tables

**Table 1.** Demographic details of patients analyzed in the present study (as obtained from the cBioportal site)

Characteristic	No.
Sex	
Male	386
Female	142
Mutation count	6‒3,181
Diagnosis age (y)	19-90
Smoking status	
Smokers	515
Data not available	12
Unknown	1
Alcohol history	
Yes	352
No	165
Data not available	11
Neoplasm histologic grade	
Grade 1	63
Grade 2	311
Grade 3	125
Grade 4	7
Grade GX	18
Data not available	4
Race category	
White	452
African	48
Asian	11
American Indian or Alaska native	2
Data not available	15

**Table 2.** The list of genes, proteins encoded, genetic alterations, loci, frequency of alteration, and variant allele frequency in genes of the *Wnt* signaling pathway

Gene	Protein	Alteration	Loci	% of alteration	Variant allele frequency in tumor sample	gnomAD data
*WNT1*	Wnt family member 1	Deep deletion	12q13.12	0.6		
		D49N			0.16	Novel
*WNT2*	Wnt family member 2	Gene amplification	7q31.2	1.6		
		Deep deletion				
		S158R			0.42	Novel
		T315P			0.23	Novel
		T315N			0.57	Novel
*WNT2B*	Wnt family member 2B	Deep deletion	1p13.2	2		
		R16L			0.63	Novel
*WNT3*	Wnt family member 3	Gene amplification	17q21.31‒q21.32	1		
		R85Q			0.68	rs1483494147
		E2Q			0.18	Novel
		A261P			0.46	Novel
		G318D			0.21	Novel
*WNT3A*	Wnt family member 3A	Gene amplification	1q42.13	1.2		
		P283L			0.33	Novel
*WNT4*	Wnt family member 4	Gene amplification	1p36.12	0.6		
		E76Q			0.26	Novel
		S176Efs*12			0.13	Novel
*WNT5A*	Wnt family member 5A	Deep deletion	3p14.3	1.4		
		I93V			0.28	rs750646727
		G341S			0.20	rs1370251695
		R215C			0.57	Novel
*WNT5B*	Wnt family member 5B	Gene amplification	12p13.33	3		
		Deep deletion				
		G242W			0.36	Novel
*WNT6*	Wnt family member 6	Deep deletion	2q35	1.8		
		R46W			0.29	rs766635655
		T105M			0.23	rs759013954
*WNT7A*	Wnt family member 7A	Gene amplification	3p25.1	0.8		
		Deep deletion				
		R90C			0.28	rs751362548
*WNT7B*	Wnt family member 7B	Gene amplification	22q13.31	0.8		
		Q261E			0.10	Novel
*WNT8A*	Wnt family member 8A	Deep deletion	5q31.2	0.6		
		G12A			0.25	Novel
		L346V			0.32	Novel
*WNT8B*	Wnt family member 8B	Deep deletion	10q24.31	1.4		
		W63C			0.36	Novel
		R157Q			0.34	Novel
		A59S			0.12	Novel
		A94S			0.02	Novel
*WNT9A*	Wnt family member 9A	Gene amplification	1q42.13	2.2		
		R333Q			0.17	Novel
		D156N			0.25	Novel
		E37K			0.41	Novel
		V290M			0.18	Novel
		R61W			0.14	Novel
		A264V			0.28	Novel
*WNT9B*	Wnt family member 9B	Gene amplification	17q21.32	0.8		
		P35A			0.20	Novel
		Y353H			0.15	Novel
		L102P			0.18	Novel
*WNT10A*	Wnt family member 10A	Deep deletion	2q35	1.8		
		A240V			0.16	rs201578578
		G88D			0.28	Novel
*WNT10B*	Wnt family member 10B	Gene amplification	12q13.12	0.4	-	-
		Deep deletion				
*WNT11*	Wnt family member 11	Gene amplification	11q13.5	5		
		Deep deletion				
		L65P			0.61	rs1173549137
		R202H			0.07	rs374455490
*WNT16*	Wnt family member 16	Gene amplification	7q31.31	1.6		
		Deep deletion				
		E271Q			0.20	Novel
		S218*			0.18	Novel
		G304W			0.09	Novel

**Table 3.** Expression profile of genes interacting with *Wnt11* in head and neck squamous cell carcinoma patients

Gene	Protein	Expression profile	Expression score (p-value)
*FZD1*	Frizzled-1; receptor for Wnt proteins	Upregulated^a^	2.237 × 10^-9^
*FZD2*	Frizzled-2; receptor for Wnt proteins	Upregulated^a^	<10^-12^
*FZD3*	Frizzled-3; receptor for Wnt proteins	Upregulated	1.372 × 10^-1^
*FZD4*	Frizzled-4; receptor for Wnt proteins	Downregulated^a^	3.639 × 10^-2^
*FZD6*	Frizzled-6; receptor for Wnt proteins	Upregulated^a^	1.624 × 10^-12^
*FZD7*	Frizzled-7; receptor for Wnt proteins	Upregulated^a^	8.622 × 10^-2^
*FZD8*	Frizzled-8; receptor for Wnt proteins	Upregulated^a^	2.518 × 10^-3^
*DVL1*	Segment polarity protein dishevelled homolog DVL-1	Upregulated^a^	4.600 × 10^-2^
*DVL2*	Segment polarity protein dishevelled homolog DVL-2	Upregulated^a^	1.624 × 10^-12^
*DVL3*	Segment polarity protein dishevelled homolog DVL-3	Upregulated^a^	<10^-12^

a Differentially expressed genes with a statistically significant gene expression.

**Table 4.** Functional enrichment of the *Wnt11* interacting genes based on the KEGG pathway analysis

KEGG pathways	Strength	False discovery rate
Basal cell carcinoma	2.49	1.76 × 10^-26^
Melanogenesis	2.3	8.06 × 10^-25^
Signaling pathways regulating pluripotency stem cells	2.15	1.93 × 10^-23^
Wnt signaling pathway	2.14	2.11 × 10^-23^
Gastric cancer	2.12	2.26 × 10^-23^
Breast cancer	2.12	2.26 × 10^-23^
mTOR signaling pathway	2.12	2.26 × 10^-23^
Cushing’s syndrome	2.11	2.26 × 10^-23^
Hippo signaling pathway	2.11	2.26 × 10^-23^
Hepatocellular carcinoma	2.08	3.38 × 10^-23^
Notch signaling pathway	2.05	3.07 × 10^-6^
HTLV-1 infection	1.89	2.96 × 10^-21^
Proteoglycans of cancer	1.86	2.27 × 10^-14^
Human papillomavirus infection	1.79	3.50 × 10^-20^
Pathways in cancer	1.58	6.22 × 10^-18^

KEGG, Kyoto Encyclopedia of Genes and Genomes; mTOR, mammalian target of rapamycin.

## References

[1] Sengupta A (2012). Recent advances in head and neck cancer. Apollo Med.

[2] Bray F, Ferlay J, Soerjomataram I, Siegel RL, Torre LA, Jemal A (2018). Global cancer statistics 2018: GLOBOCAN estimates of incidence and mortality worldwide for 36 cancers in 185 countries. CA Cancer J Clin.

[3] Szyfter K, Kiwerska K, Wierzbicka M (2018). HPV-related HNC: new challenge and hope for head and neck cancer subjects. J Med Sci.

[4] Fitzmaurice C, Allen C, Barber RM, Barregard L, Bhutta ZA, Global Burden of Disease Cancer Collaboration (2017). Global, regional, and national cancer incidence, mortality, years of life lost, years lived with disability, and disability-adjusted life-years for 32 cancer groups, 1990 to 2015: a systematic analysis for the global burden of disease study. JAMA Oncol.

[5] Gupta B, Johnson NW, Kumar N (2016). Global epidemiology of head and neck cancers: a continuing challenge. Oncology.

[6] Yan K, Agrawal N, Gooi Z (2018). Head and neck masses. Med Clin North Am.

[7] MacDonald BT, Tamai K, He X (2009). Wnt/beta-catenin signaling: components, mechanisms, and diseases. Dev Cell.

[8] Takahashi-Yanaga F, Kahn M (2010). Targeting Wnt signaling: can we safely eradicate cancer stem cells?. Clin Cancer Res.

[9] Novellasdemunt L, Antas P, Li VS (2015). Targeting Wnt signaling in colorectal cancer: a review in the theme: cell signaling: proteins, pathways and mechanisms. Am J Physiol Cell Physiol.

[10] Noguti J, CF DEM, Hossaka TA, Franco M, Oshima CT, Dedivitis RA (2012). The role of canonical WNT signaling pathway in oral carcinogenesis: a comprehensive review. Anticancer Res.

[11] Ozbey U, Attar R, Romero MA, Alhewairini SS, Afshar B, Sabitaliyevich UY (2018). Apigenin as an effective anticancer natural product: spotlight on TRAIL, WNT/beta-catenin, JAK-STAT pathways, and microRNAs. J Cell Biochem.

[12] Javed Z, Muhammad Farooq H, Ullah M, Zaheer Iqbal M, Raza Q, Sadia H (2019). Wnt signaling: a potential therapeutic target in head and neck squamous cell carcinoma. Asian Pac J Cancer Prev.

[13] Cerami E, Gao J, Dogrusoz U, Gross BE, Sumer SO, Aksoy BA (2012). The cBio cancer genomics portal: an open platform for exploring multidimensional cancer genomics data. Cancer Discov.

[14] Gao J, Aksoy BA, Dogrusoz U, Dresdner G, Gross B, Sumer SO (2013). Integrative analysis of complex cancer genomics and clinical profiles using the cBioPortal. Sci Signal.

[15] Karczewski KJ, Francioli LC, Tiao G, Cummings BB, Alfoldi J, Wang Q (2020). The mutational constraint spectrum quantified from variation in 141,456 humans. Nature.

[16] Szklarczyk D, Gable AL, Lyon D, Junge A, Wyder S, Huerta-Cepas J (2019). STRING v11: protein-protein association networks with increased coverage, supporting functional discovery in genome-wide experimental datasets. Nucleic Acids Res.

[17] Chandrashekar DS, Bashel B, Balasubramanya SA, Creighton CJ, Ponce-Rodriguez I, Chakravarthi B (2017). UALCAN: a portal for facilitating tumor subgroup gene expression and survival analyses. Neoplasia.

[18] Barker N, Clevers H (2006). Mining the Wnt pathway for cancer therapeutics. Nat Rev Drug Discov.

[19] Katoh M, Katoh M (2007). WNT signaling pathway and stem cell signaling network. Clin Cancer Res.

[20] Rao TP, Kuhl M (2010). An updated overview on Wnt signaling pathways: a prelude for more. Circ Res.

[21] Logan CY, Nusse R (2004). The Wnt signaling pathway in development and disease. Annu Rev Cell Dev Biol.

[22] Leethanakul C, Patel V, Gillespie J, Pallente M, Ensley JF, Koontongkaew S (2000). Distinct pattern of expression of differentiation and growth-related genes in squamous cell carcinomas of the head and neck revealed by the use of laser capture microdissection and cDNA arrays. Oncogene.

[23] Rhee CS, Sen M, Lu D, Wu C, Leoni L, Rubin J (2002). Wnt and frizzled receptors as potential targets for immunotherapy in head and neck squamous cell carcinomas. Oncogene.

[24] Felthaus O, Ettl T, Gosau M, Driemel O, Brockhoff G, Reck A (2011). Cancer stem cell-like cells from a single cell of oral squamous carcinoma cell lines. Biochem Biophys Res Commun.

[25] J VP, A P (2020). Virtual screening of mutations in antioxidant genes and its putative association with HNSCC: an in silico approach. Mutat Res.

[26] Anita R, Paramasivam A, Priyadharsini JV, Chitra S (2020). The m6A readers YTHDF1 and YTHDF3 aberrations associated with metastasis and predict poor prognosis in breast cancer patients. Am J Cancer Res.

